# Utility of viscoelastic hemostatic assay to guide hemostatic resuscitation in trauma patients: a systematic review

**DOI:** 10.1186/s13017-022-00454-8

**Published:** 2022-09-13

**Authors:** Zhe Zhu, Yong Yu, Kairui Hong, Mengqin Luo, Yefang Ke

**Affiliations:** 1 Department of Blood Transfusion, Hwa Mei Hospital, University of Chinese Academy of Sciences, Ningbo, Zhejiang People’s Republic of China; 2 Ningbo Institute of Life and Health Industry, University of Chinese Academy of Sciences, Ningbo, People’s Republic of China; 3 Department of Clinical Laboratory, Ningbo Women and Children’s Hospital, Liuting street 339, Ningbo, Zhejiang People’s Republic of China

**Keywords:** Viscoelastic hemostatic assay, Thrombelastography, Rotational thromboelastometry, Hemostatic resuscitation, Blood transfusion, Trauma

## Abstract

**Objective:**

Viscoelastic hemostatic assay (VHA) provides a graphical representation of a clot’s lifespan and reflects the real time of coagulation. It has been used to guide trauma resuscitation; however, evidence of the effectiveness of VHAs is still limited. This systematic review aims to summarize the published evidence to evaluate the VHA-guided strategy in resuscitating trauma patients.

**Methods:**

The PubMed, Embase, and Web of Science databases were searched from their inception to December 13, 2021. Randomized controlled trials (RCTs) or observational studies comparing VHA-guided transfusion to controls in resuscitating trauma patients were included in this systematic review.

**Results:**

Of the 7743 records screened, ten studies, including two RCTs and eight observational studies, met the inclusion criteria. There was great heterogeneity concerning study design, enrollment criterion, VHA device, VHA-guided strategy, and control strategy. Thrombelastography (TEG) was used as a guiding tool for transfusion in eight studies, and rotational thromboelastometry (ROTEM), and TEG or ROTEM were used in the other two studies. The overall risk of bias assessment was severe or mild in RCTs and was severe or moderate in observational studies. The main outcomes reported from the included studies were blood transfusion (*n* = 10), mortality (*n* = 10), hospital length of stay (LOS) (*n* = 7), intensive care unit LOS (*n* = 7), and cost (*n* = 4). The effect of the VHA-guided strategy was not always superior to the control. Most of the studies did not find significant differences in the transfusion amount of red blood cells (*n* = 7), plasma (*n* = 5), platelet (*n* = 7), cryoprecipitate/fibrinogen (*n* = 7), and mortality (*n* = 8) between the VHA-guided group and control group. Notable, two RCTs showed that the VHA-guided strategy was superior or equal to the conventional coagulation test-guided strategy in reducing mortality, respectively.

**Conclusion:**

Although some studies demonstrated VHA-guided strategy probable benefit in reducing the need for blood transfusion and mortality when resuscitating trauma patients, the evidence is still not robust. The quality of evidence was primarily downgraded by the limited number of included studies and great heterogeneity and severe risk of bias in these. Further studies are strongly recommended.

**Supplementary Information:**

The online version contains supplementary material available at 10.1186/s13017-022-00454-8.

## Introduction

Trauma is one of the major causes of death [1, 2], and it is estimated that one person will die every three minutes due to trauma globally [2]. Bleeding is responsible for approximately 25–50% of trauma deaths [1, 3]. Trauma-induced coagulopathy (TIC) is closely related to bleeding and is present in approximately 25% of severely injured trauma patients on admission [1, 4].

Hemorrhage is a main preventable cause of death in severely injured trauma patients [1, 4]. In 2007, a combat support hospital found that a higher ratio of plasma to red blood cells (RBCs) was independently associated with increased survival [5]. Then, transfused plasma and RBCs at a high, fixed ratio was introduced into damage control resuscitation (DCR) [6]. However, there are still some controversies regarding this method of treatment. First, which ratio is better? The PROPPR trial reported that mortality was not significantly different when plasma, platelets, and RBCs were administered at ratios of 1:1:1 and 1:1:2 [7]. Second, a few observational studies found that the RBCs to plasma ratio was not associated with mortality in some specific injured patients [8, 9]. Third, the characteristics and severities of patient injuries differed, and the fixed-ratio strategy may not be suitable for every injured patient.

**Fig. 1:**
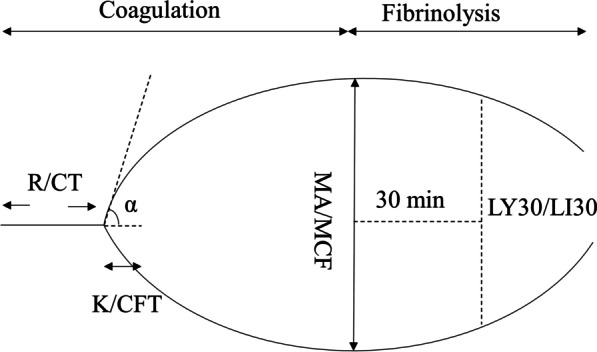
TEG/ROTEM trace. The main parameters in TEG are R, K, α-angle, MA, and LY30. The main parameters in ROTEM are CT, CFT, α-angle, MCF, and LI30. R/CT: the time from start to initial clot formation (to 2 mm amplitude), reflect coagulation function. K/CFT: the time when amplitude raises from 2 to 20 mm, reflects fibrin formation and cross-linking. α-angle: the angle between the midline and the tangent of the curve, its significance is similar to K/CFT. MA/MCF: the peak amplitude of the curve, mainly represents the platelet function. LY30/LI30: percent amplitude reduction at 30 min after MA/MCF, is a reflection of fibrinolysis

Laboratory-guided or goal-directed management is an alternative strategy. A comparison of empiric versus conventional coagulation test (CCT)-guided management in resuscitating severely injured trauma patients was performed in a randomized controlled trial (RCT) over a decade ago [10]. Compared with the fixed ratio (1:1:1), there was a declining tendency of 28-day mortality in the CCT-guided group, although no significant difference was discovered [10]. Another laboratory-guided or goal-directed management was based on viscoelastic hemostatic assay (VHA). VHA, which includes thrombelastography (TEG) and rotational thromboelastometry (ROTEM), is performed in whole blood and provides a graphical representation of a clot’s lifespan in real time, from clot formation and stabilization to lysis, which more accurately reflects hemostasis in vivo [11] (Fig. 1). In addition, it is easy to use by non-laboratory personnel [12].

VHA has been widely used to monitor the real-time coagulation status of trauma patients [11]. It has demonstrated a potential role in predicting mortality [13, 14], massive transfusion [15, 16], thrombotic events [17, 18], and guiding therapy [19]. To this end, some observational studies have compared blood product usage and safety outcomes before and after implementing a VHA-guided strategy, with different results reported [20–27]. Meanwhile, two RCTs observed that the VHA strategy had beneficial [28] or no effect [29] in reducing mortality compared to the CCT strategy. Another common problem with the published studies on VHA is the lack of justification for how TEG or ROTEM can affect outcomes and how other more important determinants (such as head injury, major injuries, age, etc.) are accounted for.

To date, there is still limited information; therefore, we performed a systematic review to assess the efficacy and safety of the VHA-guided strategy in resuscitating trauma patients by analyzing blood product usage, mortality, hospital length of stay (LOS), intensive care unit (ICU) LOS, cost, and serious adverse events (such as thromboembolic events, sepsis, and acute kidney injury).

## Methods

### Study design and search strategy

This systematic review was conducted according to the Preferred Reporting Items for Systematic Reviews and Meta-Analysis (PRISMA) statement. The PubMed, Embase, and Web of Science databases were searched from their inception to December 13, 2021. The literature was searched following the combination of “thrombelastography or rotational thrombelastometry or viscoelastic hemostatic assay” and “trauma.” Besides, reference lists from the relevant articles were also screened manually. The detailed search strategy is shown in Additional file 1: Table S1. Two reviewers (ZZ and YK) independently searched, selected studies, extracted data, and assessed study quality. Disagreements between reviewers were addressed by consulting a third reviewer (YY).

### Inclusion criteria

Studies were included if they were RCTs or observational studies that compared hemostatic resuscitation guided by TEG, ROTEM, or both to hemostatic resuscitation guided by CCT, clinical judgment, massive transfusion protocol (MTP), or pre-TEG/ROTEM protocol in adult trauma patients. Duplicate studies, review articles, case series, editorials, letters, conference meeting abstracts, articles with no definite VHA-guided strategy, and articles with no control group were excluded.

### Data extraction

Data on study design, publication year, setting, sample size, criteria of enrolled patients, VHA device, VHA-guided strategy, control strategy, and the main efficacy and safety outcomes were extracted. The efficacy and safety outcomes investigated in this study were RBCs, plasma, platelet, cryoprecipitate/fibrinogen transfusion amount, mortality, hospital LOS, ICU LOS, cost, and serious adverse events (such as thromboembolic events, sepsis, and acute kidney injury).

### Assessment of methodologic quality

The risk of bias in RCTs and observational studies was assessed by using the Cochrane risk-of-bias tool and risk of bias in non-randomised studies of interventions (ROBINS-I) tool, respectively. Additionally, the methodological qualities of observational studies were also investigated by using Newcastle–Ottawa Scale (NOS).

## Results

### Search results and characteristics of included studies

**Fig. 2:**
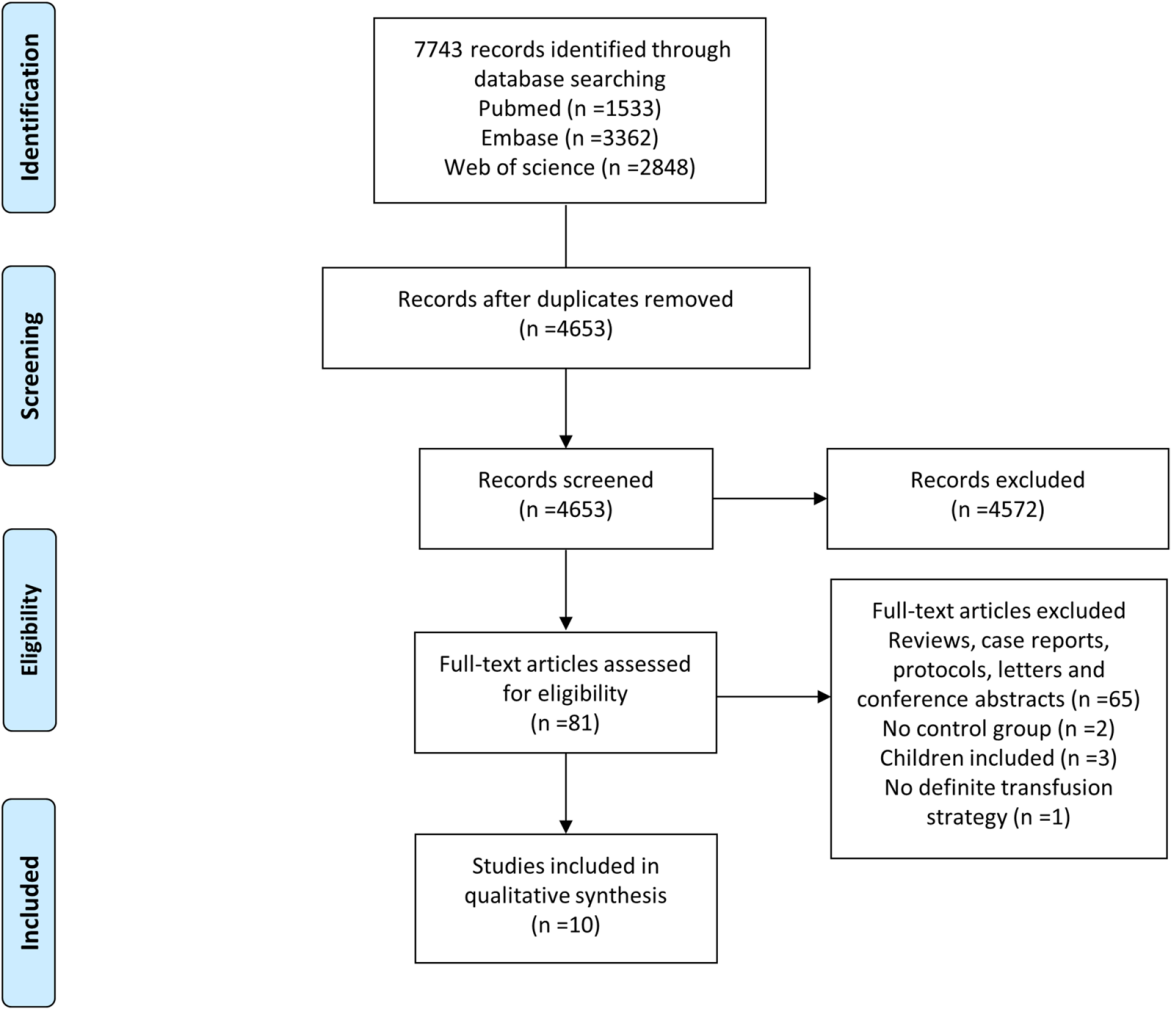
Flow diagram of study selection

Of the 7743 records screened, ten studies, including two RCTs [28, 29] and eight observational studies [20–27], were included in this systematic review (Fig. 2). Among the eight observational studies, seven were before-after comparisons [20–22, 24–27], and one was a retrospective analysis [23]. The participants in these studies were all civilian trauma patients. One study incorporated ROTEM into DCR to resuscitate combat causalities; however, it was excluded as it did not have a definite transfusion strategy [30]. Of the ten included studies, only one was a multi-center trial [29], and the remaining were single-center studies [20–28]. Most of the studies were performed in the USA [22–24, 26–28], and the others were conducted in Europe [20, 29], Australia [21], and China [25]. One center, Denver Health Medical Center, published two studies: one was a before-after observational study [27], and the other was an RCT [28]. TEG was the most frequently used VHA device in the included studies (eight studies) [20, 22–28]; ROTEM [21], and TEG or ROTEM [29] were conducted in the other two studies. Regarding the transfusion strategy, two RCTs compared TEG or ROTEM-guided strategy to CCT-guided strategy [28, 29]; eight observational studies compared TEG or ROTEM-guided strategy to non-TEG or non-ROTEM-guided strategy [20–27], two of them were CCT-based [20, 22], one was an MTP protocol [26], and the others did not mention (Table 1). The detailed transfusion strategies and relevant VHA tests are shown in Additional file 1: Table S2 and Additional file 1: Table S3. The risk of bias in RCTs is shown in Additional file 1: Figure S1; one study [28] may had more risk of bias than another [29]. The overall risk of bias assessment of observational studies was moderate or severe (Additional file 1: Table S4), and their NOS scores ranged from 6 to 7 (Additional file 1: Table S5).

**Table 1:** Characteristics of included studies

Study	Study design	Setting	Included criteria	VHA device	VHA/total sample size	Transfusion algorithm, VHA versus control group
Baksaas-Aasen [29]	Multi-center, randomized controlled trial	Seven major trauma centers in Europe	Adult trauma patients, presented with clinical signs of bleeding, activating the local MHP and if RBCs transfusion had been initiated	TEG 6S or ROTEM	201/396	TEG or ROTEM versus CCT
Cochrane [20]	Single-center, before-after study	A level I trauma center in the UK	Major hemorrhage protocol activation, with suspicion of significant active bleeding, and blood transfusion commenced	TEG 6S	175/301	Post-TEG versus pre-TEG (CCT-based)
Campbell [21]	Single-center, before-after study	A trauma center in Southeast Queensland, Australia	Adult trauma patients (≥ 18 years or older), ISS ≥ 12, and received blood products	ROTEM	77/110	Post-ROTEM versus pre-ROTEM
Unruh [22]	Single-center, before-after study	A level I trauma center in Wichita, KS, USA	All trauma patients who underwent MTP activation	TEG 5000	47/67	Post-TEG versus pre-TEG (CCT-based)
Wang [23]	Single-center, retrospective study	A level I trauma center in Fort Worth, Texas, USA	Patients who sustained traumatic liver and/or spleen injuries and received any types of blood products within the first 24 h of hospital arrival	TEG	86/166	TEG versus non-TEG
Mohamed [24]	Single-center, before-after study	A level I trauma center in Flint, MI, USA	Aged 15 years or older with an ISS of ≥ 15, and transfused within the first 24 h of presentation	TEG	47/134	Post-TEG versus pre-TEG
Gonzalez [28]	Single-center, randomized controlled trial	A level I trauma center in Denver, USA	Injured patients at least 18 years of age that met criteria for MTP activation upon ED arrival	TEG 5000	56/111	TEG versus CCT
Yin [25]	Single-center, before-after study	The ED in Nanjing, China	Older than 18 years, abdominal abbreviated injury scale ≥ 2, and requirement of 2 or more units of RBCs transfusion within 24 h of ED admission	TEG 5000	29/60	Post-TEG versus pre-TEG
Tapia [26]	Single-center, before-after study	A level I trauma center in Houston, Texas, USA	Trauma patient patients receiving 6 units or more, and 10 units or more RBCs in the first 24 h, aged ≥ 15 years	TEG	165/289(≥ 6U RBCs); 98/163(≥ 10U RBCs)	TEG versus MTP
Kashuk [27]	Single-center, before-after study	A level trauma center in Denver, Colorado, USA	Patients who received 6 or more units of blood within the first 6 h	TEG 5000	34/68	Post-TEG versus pre-TEG

*MHP* major hemorrhage protocol; *RBCs* red blood cells; *ISS* injury severity score; *MTP* massive transfusion protocol; *ED* emergency department; *VHA* viscoelastic hemostatic assay; *TEG* thrombelastography; *ROTEM* rotational thromboelastometry; *CCT* conventional coagulation tests

### Effect of the VHA-guided strategy on blood transfusion in trauma patients

Eight studies gave detailed RBCs, plasma, platelet, and cryoprecipitate/fibrinogen amounts for the included patients [20–25, 28, 29]. One study grouped the patients according to RBCs transfusion amounts (≥ 6 U or ≥ 10 U) and mechanism of injury (MOI) (blunt or penetrating) and performed a stratified analysis [26]. In the last study, the blood products were illustrated in a figure, and the data could not be extracted; it concluded that there was a trend of fewer products in the post-TEG group but did not reach a significant difference [27] (Table 2).

**Table 2:** Effect of VHA-guided strategy on blood transfusion

Study	Amounts of RBCs (Units)	Amounts of plasma (Units)	Amounts of platelets (Units)	Amounts of cryoprecipitate/fibrinogen (Units)	Main findings
	VHA group versus Control group	VHA group versus Control group	VHA group versus Control group	VHA group versus Control group	
Baksaas-Aasen [29]	6 (3–10) versus 6 (4–6)	6 (3–10) versus 7 (4–11) ^a*^	2 (1–3) versus 1 (0–2) pools ^b*^	4 (0–4) versus 3 (0–4) g ^c^	Patients in the VHA group received more platelet, and less plasma at 24 h after injury, no statistically differences in the amounts of RBCs and fibrinogen equivalent were found between the two groups Patients in the VHA group received more platelet, and fibrinogen equivalent between the baseline and hemostasis, no statistically differences in the amounts of RBCs and plasma were found between the two groups
Cochrane [20]	3.9 ± 4.0 versus 3.6 ± 3.3	2.6 ± 3.8 versus 2.5 ± 3.4 ^a^	0.4 ± 0.8 versus 0.2 ± 0.5	0. 5 ± 1.1 versus 0.3 ± 1.0 ^d^	No statistical differences in the number of blood components were found between the two groups, although there was a trend for more use of platelets in the post-TEG group
Campbell [21]	5.36 ± 5.50 versus 4.57 ± 3.77	0 ± 0.97 versus 2.19 ± 3.17 ^***^	0.51 ± 1.11 versus 0.30 ± 0.77	0.73 ± 1.68 versus 0 ± 0 g ^e ***^ 9.17 ± 14.83 versus 1.35 ± 3.46 ^d***^	RBCs amounts did not significantly change There was a significant increase in cryoprecipitate and fibrinogen in the post-ROTEM group, accompanied by a reduction in the use of plasma and prothrombinex Platelet usage was higher in the post-ROTEM group but did not reach statistical significance
Unruh [22]	6 (3–10) versus 11 (8–13) ^***^	4.5 (2–7.5) versus 4 (3–8.5)	1.5 (1–3) versus 2 (1–2)	1 (1–1) versus 2 (1-n/a) ^d^	ITT analysis demonstrated a significant reduction in the amounts of RBCs transfusions, the number of patients receiving plasma and platelets in the post-TEG group
Wang [23]	4 ± 7 versus 9 ± 10 ^**^	1 ± 5 versus 5 ± 6 ^**^	0.4 ± 1.5 versus 2.9 ± 4.8 ^**^	0.1 ± 0.5 versus 0.3 ± 1.2 ^d^	Patients in the TEG group received fewer amounts of RBCs, plasma, and platelets
Mohamed [24]	4.09 versus 7.69 ^**^	4.30 versus 6.43 ^*^	2.28 versus 0.83 ^*^	0.38 versus 0.47 ^d^	Over 24 h, all patients in the post-TEG group had less RBCs and plasma, and more platelets In the first 4 h, all patients in the post-TEG group had more plasma and platelets, and similar RBCs
Gonzalez [28]	9.5 (5–16) versus 11 (6–16)	5.0 (3–9) versus 6.0 (4–9)	1.0 (0–2) versus 1.0 (0–2)	0.0 (0–2) versus 1.0 (0–2) ^d*^	Less cryoprecipitate was used cumulatively at 24 h in the TEG group TEG group patients received less plasma, and platelets in the first 2 h of resuscitation
Yin [25]	5 (3–13) versus 6.5 (4–14)	5.7 (3.4–10) versus 6.1 (4–10.7)	0 (0–0) versus 0 (0–10)	0 (0–5) versus 0 (0–10) ^d^	No statistical differences in the amounts of blood components were found between the two groups Subgroup analysis including patients with ISS ≥ 16 showed that patients in the post-TEG group had significantly fewer consumption of RBCs, plasma, and total blood products
Tapia [26]	–	–	–	–	For patients receiving 6U or more RBCs, there was no difference in amounts of blood components between the TEG-guided group and MTP group Blunt MOI patients who received 10U or more RBCs in the TEG-guided group received less plasma
Kashuk [27]	–	–	–	–	Although there was a trend for fewer products in the post-TEG group, there were no significant differences

Data in studies of Cochrane [20], Campbell [21], and Wang [23] were expressed as mean ± standard deviation; data in studies of Mohamed [24] were expressed as mean; data in studies of Baksaas-Aasen [29], Unruh [22], Gonzalez [28], and Yin [25] were expressed as median (interquartile range). If there were data in several phases after injury, only the data in 24 h after admission/injury were recorded

*VHA* viscoelastic hemostatic assay; *TEG* thrombelastography; *ROTEM* rotational thromboelastometry; *MTP* massive transfusion protocol; *ISS* injury severity score; *RBCs* red blood cells; *MOI* mechanism of injury; ITT: intent-to-treat

^***^P < 0.001 compared with VHA group, ^**^P < 0.01 compared with VHA group, ^*^P < 0.05 compared with VHA group

^a^fresh-frozen plasma/octaplasma

^b^one pool = four individual platelet units

^c^fibrinogen equivalent

^d^cryoprecipitate

^e^fibrinogen concentrate, –: not reported

In the included studies, most of them did not find significant differences between the VHA-guided strategy and control strategy on the amount of RBCs transfusion (seven studies) [20, 21, 25–29], plasma transfusion (five studies) [20, 22, 25, 27, 28], platelet transfusion (seven studies) [20–22, 25–28], and cryoprecipitate/fibrinogen transfusion (seven studies) [20, 22–25, 27, 29].

In addition, three studies supported that the VHA-guided strategy could reduce the use of RBCs in trauma patients [22–24]. As for plasma transfusion, four studies indicated that the VHA-guided strategy reduced plasma transfusion amounts [21, 23, 24, 29]. Another study showed that blunt MOI patients who received ≥ 10 U RBCs in the TEG-guided group received less plasma than those in the MTP group, but did not find significant differences in the other patients [26]. Regarding platelet transfusion, two studies, including an RCT [24, 29], demonstrated a significant increase in platelet amount when using the VHA-guided strategy; however, one study observed the opposite result [23]. Finally, one observational study [21] and an RCT [28] observed that the VHA-guided strategy increased or decreased cryoprecipitate/fibrinogen products, respectively.

Three studies also noticed that the VHA-guided strategy had a time effect. In a before-after study, Mohamed et al. [24] observed that compared with the pre-TEG group, patients in the post-TEG group had less plasma over 24 h, but they received more in the first 4 h. In contrast, an RCT revealed that TEG group patients received less plasma and platelets in the first 2 h of resuscitation, but showed no significant differences over 24 h compared with the CCT group [28]. Meanwhile, compared with the CCT group, patients in the VHA group received more fibrinogen equivalent and similar plasma between baseline and hemostasis, but they transfused similar fibrinogen equivalent and less plasma at 24 h after injury, as revealed by the ITACTIC study [29].

### Effect of the VHA-guided strategy on mortality, hospital, and ICU LOS, and other outcomes in trauma patients

Mortality reported in the enrolled study included 6-h, 24-h, 28-day, 30-day, 90-day, and in-hospital mortality (Table 3). Overall, two studies, including an RCT, showed that the VHA-guided strategy reduced mortality among trauma patients [20, 28]. Cochrane et al. [20] found that the 24-h and 30-day mortalities were significantly lower in the post-TEG group. In addition, Gonzalez et al. [28] noticed that the 6-h and 28-day mortality rates in the TEG group were significantly lower than those in the CCT group. Overall, no significant differences were observed in mortality between the VHA-guided group and control group in the remaining eight studies, including an RCT (ITACTIC) [21–25, 27, 29]. However, in the ITACTIC study, a reduction in 28-day mortality was observed in trauma patients who also had a severe traumatic brain injury (TBI) when guided by VHA [29]. In another study, total mortality was significantly reduced in trauma patients < 30 years after implementing a TEG strategy, although no difference was found in all trauma patients [24]. Besides, Tapia et al. found that the TEG-directed resuscitation decreased 30-day mortality in penetrating trauma patients who received 10 U or more RBCs, but showed no effect on the other trauma patients [26].

**Table 3:** Effect of VHA-guided strategy on mortality and other outcomes

Study	Mortality (%)	Main findings in the effect of transfusion strategy on mortality	Main findings in the effect of transfusion strategy on the other outcomes
	VHA group versus control group		
Baksaas-Aasen [29]	11% versus 11% ^a^ 14% versus 17% ^b^ 25% versus 28% ^c^ 29% versus 31% ^d^ 25% versus 30% ^e^	There were no statistical differences in mortality in each phase 28-day mortality was reduced in the patients who also had severe TBI in the VHA group	There were no statistically significant differences in other outcomes between the two study groups, including the proportion of patients who were alive and free of massive transfusion, rate of multiple organ dysfunction, the incidence of symptomatic thromboembolic events, 28-day ventilator-free days or ICU-free days, and hospital LOS. So did the serious adverse events More patients in the VHA group received a study intervention before hemostasis, and at 24 h after injury The study interventions were given a median of 21 min earlier in the VHA group
Cochrane [ 20]	5% versus 13% ^b**^ 11% versus 25% ^c**^	Mortality was significantly lower in the post-TEG group at 24 h and 30 days	Total hospital LOS was significantly greater in the post-TEG group Total cost and cost of transfusion did not reach statistically significant between the two groups Blood product wastage was significantly lower in the post-TEG group
Campbell [21]	16.9% versus 13.5% ^f^ 15.6% versus 13.5% ^g^	There were no significant differences in mortality during hospital or ICU admission	No significant difference was seen in the ICU or hospital LOS Costs of blood products were higher in the post-ROTEM group
Unruh [22]	31.9% versus 55% ^f^	A trend toward reduced mortality (*P* = 0.076) was observed in the post-TEG group, but it did not reach a significant difference	There was no significant study period effect on ICU admissions or ICU days, mechanical ventilation use, or hospital LOS A trend toward increased ICU days (11 vs. 7 days, *P* = 0.073) was observed in the post-TEG group
Wang [23]	3% versus 10% ^b^ 12% versus 19% ^f^	There were no significant differences in 24-h mortality or in-hospital total mortality	Shorter hospital and ICU LOS were found in the patients of the TEG-guided group, excluded those who died within the initial 24 h of hospital arrival
Mohamed [24]	34.04% versus 36.78% ^f^	The overall mortality rate had no significant difference between the two groups However, the mortality rate was significantly lower in patients < 30 years in the post-TEG group (pre-TEG 42.5% versus post-TEG 14.29%, *P* = 0.0451)	Patients in the post-TEG group had a shorter hospital and ICU LOS Costs of blood products were reduced in the post-TEG group, especially in patients with penetrating injuries
Gonzalez [28]	7.1% versus 21.8% ^a*^ 19.6% versus 36.4% ^c*^ 8.9% versus 20% ^e^	ITT analysis showed that the 6-h and 28-day mortality in the TEG group was significantly lower than in the CCT group There were no significant differences in hemorrhagic deaths between the two groups in the ITT analyses; however, it reached significant differences in the AT analyses	Patients in the TEG group had more ICU-free days (*P* = 0.091), and more ventilator-free days (*P* = 0.082) than those in the CCT group; however, these differences were not statistically significant The groups had similar rates of sepsis, AKI, DVT, and pulmonary embolism
Yin [25]	10.3% versus 6.5% ^c^	No significant differences were found in mortality at 28-d between the two groups	No significant differences were found in ICU and hospital LOS between the two groups Costs of blood products appeared to be lower in the TEG group but were not significantly different At 24 h, patients in the TEG group had shorter aPTT compared to patients in the control group
Tapia [26]	–	For patients who received 6U or more RBCs, and blunt trauma patients who received 10U or more RBCs, there was no difference in mortality between the TEG-guided group and MTP group While 30-day mortality decreased in penetrating trauma patients who received 10U or more RBCs in the TEG-directed group	–
Kashuk [27]	29% versus 65% ^f^	The overall mortality fell after the TEG algorithm implementation; however, it did not reach a significant difference	–

*VHA* viscoelastic hemostatic assay; *TEG* thrombelastography; *ROTEM* rotational thromboelastometry; *CCT* conventional coagulation test; *RBCs* red blood cells; *MOI* mechanism of injury; *LOS* length of stay; *ICU* intensive care unit; *TBI* traumatic brain injury; *AKI* acute kidney injury; *DVT* deep vein thrombosis; *ISS* injury severity score; *ITT* intent-to-treat; *AT* as treated; *MTP* massive transfusion protocol; *aPTT* activated partial thromboplastin time

^**^P < 0.01 compared with VHA group, ^*^P < 0.05 compared with VHA group

^a^6-h mortality

^b^24-h mortality

^c^28-day or 30-day mortality

^d^90-day mortality

^e^death from exsanguination

^f^in-hospital total mortality

^g^mortality in ICU, –: not reported

Seven studies reported on total hospital LOS [20–25, 29]. Two studies found a shorter hospital LOS [23, 24], one study observed a longer hospital LOS in the VHA-guided group [20], and the others did not find significant differences between the two groups [21, 22, 25, 29]. Seven studies described ICU LOS [21–25, 28, 29]. Two studies found a shorter ICU LOS in the VHA-guided group [23, 24], but five studies did not find any significant differences between the two groups [21, 22, 25, 28, 29] (Table 3).

Two RCTs reported that the incidences of serious adverse events, such as thromboembolic events, sepsis, and acute kidney injury, all showed similar rates [28, 29] (Table 3). In addition, four before-after observational studies performed cost analysis, with different results. They reported that the cost of blood products in the post-VHA group was higher [21], lower [24] than, or equal [20, 25] to the pre-VHA group.

## Discussion

This systematic review, including two RCTs and eight observational studies, sought to collect evidence to assess the efficacy and safety of a VHA-guided strategy in resuscitating trauma patients. The different study designs, VHA devices, VHA-guided strategies, enrollment criteria, control groups, and non-uniformity of the variables in the included studies make it impossible to combine the results and conduct a meta-analysis. We collected data on blood product amounts, mortality, hospital LOS, ICU LOS, etc., in trauma patients, compared the VHA-guided strategies to control strategies (CCT-guided, MTP, or unknown) and performed a qualitative analysis. Overall, there was no robust evidence to support that a VHA-guided strategy could decrease blood products and mortality among trauma patients.

VHA-guided transfusion was initially implemented in cardiac and liver surgery [31, 32], and it may play an important role in reducing mortality and saving blood. A systematic review and meta-analysis included 17 RCTs (15 involving cardiac surgery) and found that TEG or ROTEM was helpful in reducing overall mortality and the need for RBCs, FFP, and platelets in patients with bleeding. However, it indicated that the evidence was low quality, mainly due to few events and poorly designed trials [12]. Meanwhile, another meta-analysis, which included 9 RCTs conducted in elective surgery settings (7 cardiac surgeries and 2 liver surgeries), observed that plasma transfusion, platelet transfusion, operating room LOS, ICU LOS, and bleeding rate were reduced with TEG-guided transfusion when compared to controls; RBCs transfusion had a declining tendency in the TEG group, but did not reach statistical significance; however, mortality was comparable between the TEG and control groups [33].

Recently, a review [34] included 2 RCTs and 5 observational studies and performed a meta-analysis. It found that the TEG/ROTEM-guided strategy was associated with a tendency toward fewer blood product transfusions and a reduction in mortality in acutely bleeding trauma patients. The contradiction between Bugaev et al.’s review and our study may be related to the followings: First, observational studies are likely to have selection bias; thus, the inclusion of observational studies in the meta-analysis may bias the combined results and downgrade the quality of evidence. Second, one RCT included in Bugaev et al.’s review was performed in patients undergoing surgical excision of burn wounds, not trauma patients undergoing hemostatic resuscitation [35]. Third, an RCT published in 2021 [29] reported a different result concerning mortality rates than Bugaev et al.’s review.

To date, only two RCTs have utilized VHA to guide hemostatic resuscitation in the trauma setting. Overall, the amounts of blood components were comparable between the VHA group and CCT group in the two RCTs, except for some minor divergences [28, 29]. Over 24 h, Gonzalez et al. [28] reported that compared with the CCT group, patients in the TEG group received less cryoprecipitate and similar amounts of platelets and plasma, while the ITACTIC study demonstrated that patients in the VHA group received more platelets, less plasma, and similar fibrinogen equivalent [29]. The early phase after injury was the period when death most happened owing to exsanguination and when the survival benefit occurred [28]. Therefore, Gonzalez et al. [28] analyzed the first 2 h of resuscitation and found that TEG group patients received less plasma and platelets. In contrast, in the ITACTIC study, patients in the VHA group received more platelets and fibrinogen equivalents between baseline and hemostasis [29].

However, the major divergence between the two RCTs lies in mortality [28, 29]. By “intent-to-treat” (ITT) analysis, Gonzalez et al. [28] noticed that the 6-h and 28-day survival rates in the TEG group were significantly higher than those in the CCT group, and the difference widened in the “as-treated” (AT) analysis. However, the ITACTIC study found no significant differences between the two groups at 6 h, 24 h, 28 days, or 90 days [29]. According to Baksaas-Aasen et al., coagulation monitoring cannot alter the clinical outcome in patients who never develop coagulopathy [29]. In view of that patients in the CCT group received less empiric transfusion in Gonzalez et al.’s RCT, and the role of CCT monitoring during bleeding was not reported; Baksaas-Aasen et al. [29] deduced that this might cause decreased mortality with TEG-directed therapy in Gonzalez et al.’s RCT. On the other hand, Moore et al. reported that in the ITACTIC study, VHA transfusion thresholds were based on the same thresholds as conventional testing in the CCT group. Therefore, they indicated that the patients in the VHA and CCT groups might be treated similarly, eventually leading to the groups having the same clinical outcome [1]. In our opinion, this divergence may be due to the different transfusion strategies, VHA devices, sample sizes, clinician familiarity with the VHA strategy, clinician competence, heterogenicity of the patients, injuries, etc. The optimal transfusion strategy based on VHA needs further exploration.

The results of these eight observational studies should be interpreted with caution, mainly due to their selection bias. Mortality may be affected by sex. In the study of Cochrane et al. [20], mortalities at 24 h and 30 days were significantly lower in the post-TEG group, in the case of more males in the post-TEG group. Besides, blood usage is influenced by imbalanced physiological status and therapeutic methods. In one study, FFP and cryoprecipitate were significantly different between the pre-ROTEM and post-ROTEM groups, while the post-ROTEM group had a lower admission heart rate but a higher percentage of anticoagulant use [21]. RBCs amount and the number of patients receiving FFP and platelets were reduced in the post-TEG period in the study of Unruh et al. [22]; however, patients in the post-TEG group had higher hemoglobin and hematocrit levels. Wang et al. [23] also found a significant reduction in the amounts of RBCs, plasma, and platelets in the TEG group, but patients in the TEG group were younger, had higher initial systolic blood pressure, and had lower injury severity scores (ISS). Patients with higher admission systolic blood pressure and lower ISS in the TEG-directed group were also noted in the studies of Yin et al. [25] and Kashuk et al. [27], respectively. Besides, excessive use of crystalloids caused hemodilution, thereby exacerbating hypocoagulation [36]. However, in the study of Tapia et al., the TEG group was administered more crystalloids than the MTP group [26].

In three studies, the researchers observed that the VHA strategy did not benefit the trauma patients overall but reported that it might be helpful in the treatment of some specific groups [24, 26, 29]. Despite no significant differences being found in the overall mortality between the VHA and CCT-guided interventions in the ITACTIC study, a reduction in 28-day mortality in the VHA group was observed in patients who also had severe TBI [29]. In their view, correction of coagulopathy via VHA may reduce intracerebral bleeding, cerebral ischemia, or cerebral inflammation [29]. Besides, total mortality was significantly reduced in trauma patients < 30 years after implementing a TEG strategy, despite no difference being found in all trauma patients [24]. Meanwhile, Tapia et al. [26] observed that the TEG-directed resuscitation decreased 30-day mortality in penetrating MOI patients who received 10 U or more RBCs; however, it showed no effect on the other trauma patients. However, the small sample size in the specific groups may bias the results. Therefore, further prospective studies need to be performed to explore whether the VHA-guided transfusion strategy is more likely to benefit some specific groups.

Four before-after observational studies performed cost analyses. Two studies indicated that the cost of transfusion did not reach statistical significance between the post- and pre-TEG groups [20, 25]. While in the other studies, increased [21] and decreased [24] transfusion costs were found in the post-ROTEM and post-TEG groups, respectively; however, they did not conduct statistical analyses. A prior systematic review observed that viscoelastic testing was more cost-saving than CCTs in cardiac surgery, and it deduced a more substantial cost-saving in trauma patients [37]. As no relevant trauma RCTs performed a cost analysis, the evidence of the cost-saving guided by the VHA strategy was weak.

TIC is a complex process that includes endothelial damage, coagulation system impairment, fibrinolytic activation, platelet dysfunction, and immune function abnormal [1, 38]. As a coagulation assay, it is difficult for VHA to reflect the status of TIC completely. And although plasma can supply coagulation factors and even protect the endothelium [39, 40], goal-directed hemostatic resuscitation via VHA could not fully correct TIC.

In the included studies, four studies showed that the severity of head injury was comparable between the VHA-guided group and control group [20, 22, 28, 29]; three studies did not include head injury patients or excluded the patients deemed to have died from an associated head injury [23, 25, 27], while three studies did not mention the severity of head injury [21, 24, 26]. Only two RCTs reported the cause of death. Gonzalez et al.’s RCT observed that TBI deaths did not differ significantly between the VHA-guided group and the CCT-guided group (7.1% vs. 10.9%, *P* = 0.537). On the other hand, the ITACTIC study showed that the deaths from head injury were comparable across the two groups (30% vs. 34%). In view that the VHA-guided strategy is mainly aimed at preventing death from hemorrhage, and TBI is another major cause of death after trauma; thus, the effect of TBI should not be neglected when interpreting these results.

There are some limitations to this study. First, due to the different study design, enrolled criteria, transfusion strategies, VHA devices, control strategies, and the non-uniformity of the variables in the included studies, it was challenging to perform a meta-analysis. Second, the patients in the VHA and control groups in some observational studies were incomparable, making the results less robust. Third, it should be noted that one center published two studies. One was an observational study, one of the earliest studies on this issue [27], and another was an RCT, one of two RCTs to date [28]. This fact might affect the comprehensive results. Fourth, the number of studies used for the review was relatively low, especially since only two RCTs have been published. However, this systematic review, including published RCTs and observational studies regarding VHA-guided hemostatic resuscitation in trauma patients, may provide an idea and method to improve the current strategy for further studies.

## Conclusion

In conclusion, VHA monitors the coagulation state in real time, which allows the creation of goal-directed hemostatic resuscitation in trauma patients. We present an overview of the published studies exploring VHA-guided hemostatic resuscitation in trauma patients. The number of included studies was small, especially since there were only two RCTs, and the results varied. TEG was more used as a guiding tool for transfusion than ROTEM. Although some studies demonstrated VHA-guided strategy probable benefit in reducing the need for blood transfusion and mortality, the evidence is still not robust. The quality of evidence was primarily downgraded by the limited number of included studies, and great heterogeneity and severe risk of bias in these. More ideally, large multi-center RCTs are strongly recommended.

## Supplementary Information

**Additional file1: Table S1**. Search strategy. **Table S2**. Transfusion strategy. **Table S3**. Summary of VHA tests in this study. **Table 4**. The risk of bias in observational studies.** Table S5**. The Newcastle-Ottawa Scale of included observational studies. **Fig. S1**. The risk of bias in randomized controlled trails.

## Data Availability

All data generated or analyzed during this study are included in this published article and its supplementary information files.

## Abbreviations

VHA: Viscoelastic hemostatic assay
TEG: Thrombelastography
ROTEM: Rotational thromboelastometry
CCT: Conventional coagulation test
RCT: Randomized controlled trial
TIC: Trauma-induced coagulopathy
RBCs: Red blood cells
DCR: Damage control resuscitation
FFP: Fresh-frozen plasma
TEM: Thromboelastometry
PCC: Prothrombin complex concentrate
LOS: Length of stay
ICU: Intensive care unit
MTP: Massive transfusion protocol
NOS: Newcastle–Ottawa Scale
MOI: Mechanism of injury
