# Supplementary material for: Utility of viscoelastic hemostatic assay to guide hemostatic resuscitation in trauma patients: a systematic review
Source: World J Emerg Surg. 2022 Sep 13;17:48. doi: 10.1186/s13017-022-00454-8 (PMC9472418; doi:10.1186/s13017-022-00454-8)
Supplement: Supplementary file 1 — Additional file1: Table S1. Search strategy. Table S2. Transfusion strategy. Table S3. Summary of VHA tests in this study. Table 4. The risk of bias in observational studies. Table S5. The Newcastle-Ottawa Scale of included observational studies. Fig. S1. The risk of bias in randomized controlled trails. [file 13017_2022_454_MOESM1_ESM.docx]

**Additional Table 1. Search strategy**

| Pubmed | |
| --- | --- |
| **#1** | **"****Thrombelastography"** [MeSH Terms] |
| #2 | "thromboelasto*" [Title/Abstract] OR "thrombelasto*" [Title/Abstract] OR "TEG" [Title/Abstract] OR "rotational thromboelastometry" [Title/Abstract] OR "rotational thrombelastometry" [Title/Abstract] OR "rotational thromboelastography" [Title/Abstract] OR "rotational thrombelastography" [Title/Abstract] OR "ROTEM" [Title/Abstract] OR "viscoelastic haemostatic assay" [Title/Abstract] OR "viscoelastic hemostatic assay"[ Title/Abstract] OR "VHA" [Title/Abstract] |
| #3 | #1 OR #2 |
| #4 | "Wounds and Injuries"[MeSH Terms] |
| #5 | "trauma*"[Title/Abstract] OR "injury"[Title/Abstract] OR "injuries"[Title/Abstract] |
| #6 | #4 OR #5 |
| #7 | #3 AND #6 |
| Embase | |
| **#1** | 'thromboelastography'/exp |
| #2 | thromboelasto*:ab,ti OR thrombelasto*:ab,ti OR teg:ab,ti OR 'rotational thromboelastometry':ab,ti OR 'rotational thrombelastometry':ab,ti OR 'rotational thromboelastography':ab,ti OR 'rotational thrombelastography':ab,ti OR rotem:ab,ti OR 'viscoelastic haemostatic assay':ab,ti OR vha:ab,ti OR 'viscoelastic hemostatic assay':ab,ti |
| #3 | #1 OR #2 |
| #4 | 'injury'/exp |
| #5 | trauma*:ab,ti OR injury:ab,ti OR injuries:ab,ti |
| #6 | #4 OR #5 |
| #7 | #3 AND #6 |
| Web of science | |
| **#1** | Topic: (thromboelasto*) OR Topic: (thrombelasto*) OR Topic: (teg) OR Topic: ("rotational thromboelastometry") OR Topic: ("rotational thrombelastometry") OR Topic: ("rotational thromboelastography") OR Topic: ("rotational thrombelastography") OR Topic: (rotem) OR Topic: ("viscoelastic haemostatic assay") OR Topic: ("viscoelastic hemostatic assay") OR Topic: (VHA) |
| #2 | Topic: (injury) OR Topic: (injuries) OR Topic: (trauma*) |
| #3 | #1 and #2 |

**Additional Table 2. Transfusion strategy**

| Study | VHA tests | VHA-guided transfusion strategy | Control transfusion strategy |
| --- | --- | --- | --- |
| Baksaas‑Aasen 2021 | 1) ROTEM-guided: FIBTEM, and EXTEM  2) TEG-guided: FF TEG, and rTEG | 1) ROTEM-guided: If FIBTEM CA5 < 10mm, give additional 4g equivalent of fibrinogen (as cryoprecipitate or concentrate). If (EXTEM CA5-FIBTEM CA5) < 30mm, give 1 additional pool of platelets. If EXTEM CA5 ≥ 40 mm and EXTEM CT > 80s, give 4 additional units of plasma. If EXTEM LI30 < 85%, give additional 1 g tranexamic acid.  2) TEG-guided: If FF TEG MA < 20mm, give additional 4g equivalent of fibrinogen (as cryoprecipitate or concentrate). If (rTEG MA - FF TEG MA) < 45mm, give 1 additional pool of platelets. If rTEG MA ≥ 65 mm and rTEG ACT > 120s, give 4 additional units of plasma. If rTEG LY30 > 10%, give additional 1 g tranexamic acid. | CCT-guided: If fibrinogen < 2g/L, give additional 4g equivalent of fibrinogen (as cryoprecipitate or concentrate). If platelet < 100× 10^9^/L, give 1 additional pool of platelets. If INR > 1.2 and fibrinogen ≥ 2 g/L, give 4 additional units of plasma. |
| Cochrane 2020 | FF TEG, and rTEG | If FF TEG MA ≥ 14mm, not give cryoprecipitate; if FF TEG MA ＜14mm, and rTEG MA＜50mm, give 3 pools of cryoprecipitate; if FF TEG MA ＜14mm and rTEG MA ≥ 50mm, give 2 pools of cryoprecipitate. If rTEG ACT＜120s, not give FFP/Octoplas; if rTEG ACT ≥ 120s, give FFP/Octoplas 12-15. If rTEG MA ≥ 60mm, not give platelets; if rTEG MA ＜ 60mm, and FF TEG MA ≥ 14mm, give 1 pool of platelet; if rTEG MA＜60mm, and FF TEG MA ＜14mm, give cryoprecipitate. If LY30 ≥ 3%, give tranexamic acid 1g or 10mg/kg ; if LY30 ＜3%, not give tranexamic acid. | CCT was used to direct blood transfusion to maintain the following results: platelets > 75 × 109/L, PT/APTT < 1.5 × normal, fibrinogen > 1.5–2.0 g/L. |
| Campbell 2020 | FIBTEM, and EXTEM | ROTEM-guided: Step 1: FIBTEM CT > 600s, and EXTEM CA5 < 35mm, give tranexamic acid 1g + fibrinogen 4g; ML% > 5%, give tranexamic acid 1g. Step 2: FIBTEM CA5 ≤ 8mm, give 1 g/25 kg BW of fibrinogen; FIBTEM CA5 ≤ 10mm, give 1 Unit/5 kg BW of cryoprecipitate. Step 3: FIBTEM CA5 > 10mm and EXTEM CA5 ≤ 35mm, give 1 dose of platelet. Step 4: FIBTEM CA5 > 10mm and EXTEM CT ≥ 90s, give 10 IU/kg of PCC or 2-4 units of FFP. Step 5: To get the following targets: FIBTEM CA10 > 15mm and EXTEM CA10 > 45mm and EXTEM CT <80s. | -- |
| Unruh 2019 | -- | TEG-guided: R time >10 s = transfuse 2 units FFP, K time >3 s = transfuse 1 unit cryoprecipitate, α-angle <53° = transfuse 1 unit cryoprecipitate, MA < 50 mm = transfuse 1 unit platelet, LY30 > 3%=transfuse tranexamic acid. | -- |
| Wang 2017 | Kaolin TEG | TEG-guided: 1) FFP given when prolonged R-time (s) resulted; 2) cryoprecipitate given when prolonged K-time (s) or reduced α-angle resulted; 3) platelets given when reduced MA resulted. | -- |
| Mohamed 2017 | rTEG | TEG-guided: rTEG ACT > 128, transfuse plasma and RBCs. R-value >1.1, transfuse plasma and RBCs. K-time > 2.5, transfuse plasma, add cryoprecipitate/fibrinogen if α-angle also abnormal. α-angle < 56°, transfuse cryoprecipitate (or fibrinogen). Add platelets if MA is also abnormal. MA < 55, transfuse platelets, add cryoprecipitate/fibrinogen if α-angle also abnormal. LY30 > 3%, administer tranexamic acid or amino-caproic acid. | -- |
| Gonzalez 2016 | rTEG | TEG-guided: those patients with the first measurement of rTEG ACT ≥ 140s, received 2 plasma units, 10-pack of cryoprecipitate, and 1 unit of apheresis platelets. If the ACT was 111 to 139s, only gave 2 units of plasma. For subsequent TEGs, ACT > 110s gives 2 units of plasma, angle < 63° give 10-pack of cryoprecipitate, MA < 55 mm gives 1 apheresis platelet unit, and LY30 ≥ 7.5% gives tranexamic acid (1 g, intravenous). After August 31, 2012, the LY30 ≥ 3.0% gives tranexamic acid. | CCT-guided: INR ≥ 1.5, give 2 units of plasma. Fibrinogen < 150 mg/dL. give 10-pack of cryoprecipitate. Platelet count < 100,000/μL, give 1 unit of apheresis platelets. Suspicion of fibrinolysis with an elevated D-dimer (>0.5 μg/mL) gives tranexamic acid (1 g, intravenous). |
| Yin 2014 | Kaolin TEG | TEG-guided: If R time was 8-10 min, give 0-2U FFP; if R time was 10-12 min, give 2-4U FFP; if R time> 12 min, give 4-6U FFP. If α-angle < 60 °, give cryoprecipitate. If MA <55 mm, give PLT or rFⅦ. | -- |
| Tapia 2013 | -- | TEG-guided: If R time was 7-10 min, give 1 unit FFP or 4ml/kg, if R time was 11-14 min, give 2 units FFP or 8ml/kg, if R time > 14, give 4 units FFP or 16ml/kg. If MA was 48-54mm, give 0.3 mcg/kg DDVP, if MA was 41-48 mm, give 5 units of platelets, if MA ≤ 40 mm, give 10 units of platelets. If α-angle < 45°, give 0.6u/kg cryoprecipitate. If EPL ≥ 7.5%, give fibrinolytic of choice. | MTP protocol: on activation, 6U of type-specific RBCs, 2U of jumbo FFP (1 jumbo FFP = 2U FFP), and 1 platelet apheresis (also known as a ‘six-pack’) are prepared on receipt of a blood sample for type and crossmatch. For each subsequent request for blood products, a cooler holding 6U RBCs, 2U jumbo FFP, and 1 platelet apheresis is brought to the patient until the MTP is terminated. |
| Kashuk 2012 | rTEG | TEG-guided: rTEG G < 5.0 and patient is bleeding. If ACT > 110s, consider FFP. If rTEG ACT ≤ 110 sec and α-angle < 66°, re-check r-TEG if the patient has received FFP and is improving, consider cryoprecipitate if TEG α-angle is still < 66°. If TEG α-angle ≥ 66° and MA < 54mm, re-check r-TEG if the patient has received FFP and is improving, consider platelets + DDAVP if MA is still < 54mm. If TEG α-angle ≥ 66° and MA ≥ 54mm, consider DDAVP. | -- |

VHA: viscoelastic hemostatic assay, TEG: thrombelastography, ROTEM: rotational thromboelastometry, CCT: conventional coagulation tests, CA5: clot amplitude at 5 min, CT: clotting time, LI30: lysis index at 30 min, ML: maximun lysis, FF TEG: functional fibrinogen TEG, rTEG: rapid TEG, MA: Maximum Amplitude, R-time: reaction time, k-time: kinetic time, ACT: activated clotting time, LY30: clot lysis at 30 min, RBCs: red blood cells, FFP: fresh frozen plasma, PCC: prothrombinex, MTP: massive transfusion protocol, PT: prothrombin time, APTT: activated partial thromboplastin time, INR: international normalized ratio, BW: body weight, EPL: estimated percent lysis, DDAVP: desmopressin, --: not reported.

**Additional Table 3. Summary of VHA tests in this study**

| VHA tests | Reagents | Interpretations |
| --- | --- | --- |
| EXTEM | Tissue factor | Assess the status of extrinsic coagulation pathway. |
| FIBTEM | Cytochalasin D + tissue factor | Blocks the platelets from clot formation, thus leaving fibrinogen contributing to the strength of clot. This test could detect the status of fibrinogen. |
| Kaolin TEG | Kaolin | Assess the status of intrinsic coagulation pathway. |
| rTEG | Kaolin + tissue factor | Provides more rapid results than kaolin TEG. |
| FF TEG | Abciximab +tissue factor | Similar to FIBTEM. |

TEG: thrombelastography, rTEG: rapid TEG, FF TEG: functional fibrinogen TEG.

**Additional Table 4. The risk of bias in observational studies**

| Study | Bias due to confounding | Bias in selection of participants into the study | Bias in classification of interventions | Bias due to deviations from intended interventions | Bias due to missing data | Bias in measurement of outcomes | Bias in selection of the reported result | Overall bias |
| --- | --- | --- | --- | --- | --- | --- | --- | --- |
| Cochrane 2020 | Moderate | Low | Low | Low | Low | Moderate | Low | Moderate |
| Campbell 2020 | Moderate | Low | Low | Moderate | Low | Moderate | Low | Moderate |
| Unruh 2019 | Serious | Low | Low | Serious | Low | Moderate | Low | Serious |
| Wang 2017 | Serious | Moderate | Low | Low | Low | Moderate | Low | Serious |
| Mohamed 2017 | Moderate | Low | Low | Low | Low | Moderate | Moderate | Moderate |
| Yin 2014 | Moderate | Low | Low | Low | Low | Moderate | Low | Moderate |
| Tapia 2013 | Moderate | Low | Low | Moderate | Low | Moderate | Low | Moderate |
| Kashuk 2012 | Serious | Low | Low | Low | Low | Moderate | Low | Serious |

**Additional Table 5. The Newcastle-Ottawa Scale of included observational studies**

| Study | Patient selection | Comparability | Exposure/Outcome | NOS score |
| --- | --- | --- | --- | --- |
| Catriona Cochrane 2020 | ★☆★★ | ★☆ | ☆★★ | 6 |
| Don CAMPBELL 2020 | ★☆★★ | ★☆ | ★★★ | 7 |
| Mitchell Unruh 2019 | ★☆★★ | ★☆ | ☆★★ | 6 |
| Hao Wang 2017 | ★★★★ | ☆☆ | ★★★ | 7 |
| Mohamed Mohamed 2017 | ★☆★★ | ★☆ | ☆★★ | 6 |
| Jianyi Yin 2014 | ★☆★★ | ★☆ | ★★★ | 7 |
| Nicole M 2013 | ★☆★★ | ★☆ | ★★★ | 7 |
| Jeffry L 2012 | ★☆★★ | ★☆ | ★★☆ | 6 |


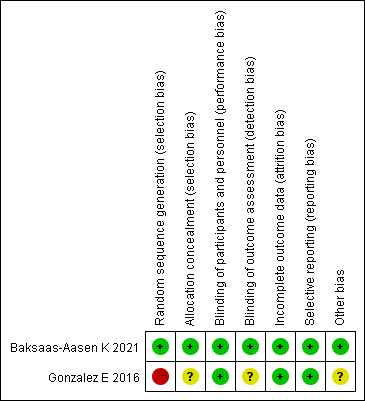


**Additional Figure 1. The risk of bias in randomized controlled trails**
